# High-performance artificial nitrogen fixation at ambient conditions using a metal-free electrocatalyst

**DOI:** 10.1038/s41467-018-05758-5

**Published:** 2018-08-28

**Authors:** Weibin Qiu, Xiao-Ying Xie, Jianding Qiu, Wei-Hai Fang, Ruping Liang, Xiang Ren, Xuqiang Ji, Guanwei Cui, Abdullah M. Asiri, Ganglong Cui, Bo Tang, Xuping Sun

**Affiliations:** 1 Institute of Fundamental and Frontier Sciences, University of Electronic Science and Technology of China, Chengdu, 610054 Sichuan China; 2 College of Chemistry, Nanchang University, Nanchang, 330031 Jiangxi China; 3 Key Laboratory of Theoretical and Computational Photochemistry, Ministry of Education, College of Chemistry, Beijing Normal University, Beijing, 100875 China; 4 College of Chemistry, Chemical Engineering and Materials Science, Shandong Normal University, Jinan, 250014 Shandong China; 5 Chemistry Department, Faculty of Science and Center of Excellence for Advanced Materials Research, King Abdulaziz University, P.O. Box 80203, Jeddah, 21589 Saudi Arabia

## Abstract

Conversion of naturally abundant nitrogen to ammonia is a key (bio)chemical process to sustain life and represents a major challenge in chemistry and biology. Electrochemical reduction is emerging as a sustainable strategy for artificial nitrogen fixation at ambient conditions by tackling the hydrogen- and energy-intensive operations of the Haber–Bosch process. However, it is severely challenged by nitrogen activation and requires efficient catalysts for the nitrogen reduction reaction. Here we report that a boron carbide nanosheet acts as a metal-free catalyst for high-performance electrochemical nitrogen-to-ammonia fixation at ambient conditions. The catalyst can achieve a high ammonia yield of 26.57 μg h^–1^ mg^–1^_cat._ and a fairly high Faradaic efficiency of 15.95% at –0.75 V versus reversible hydrogen electrode, placing it among the most active aqueous-based nitrogen reduction reaction electrocatalysts. Notably, it also shows high electrochemical stability and excellent selectivity. The catalytic mechanism is assessed using density functional theory calculations.

## Abstract (web summary)

Electrochemical reduction of nitrogen is a promising route to industrial-scale nitrogen fixation at ambient conditions, but is challenged by activation of inert nitrogen. Here the authors report a metal-free catalyst that selectively reduces nitrogen to ammonia with high efficiency and stability.

## Introduction

Ammonia (NH_3_) is an essential building block for manufacturing synthetic chemicals, such as fertilizers, medicaments, dyes, explosives, and resins^1–3^. NH_3_ has also received attention as an alternative energy carrier to advance a low-carbon society due to its large hydrogen capacity (17.6 wt%) and high energy density (4.3 kWh h^–1^)^4^. The ever-increasing demand for NH_3_ has stimulated significant research interest in artificial nitrogen (N_2_) fixation^5–13^. Currently, industrial-scale NH_3_ production mainly relies on the Haber–Bosch process at high temperature and pressure using N_2_ and hydrogen (H_2_) as feed gases^14–16^. However, this process accounts for ~2% of the worldwide energy use, i.e., 34 GJ ton_NH3_^–1^ and produces a large amount of CO_2_ (~2 ton_CO2_ ton_NH3_^–1^)^17,18^. In this regard, it is highly imperative to develop lower-energy N_2_-fixation methods, ideally operating at low temperature and pressure.

Biological N_2_ fixation is catalyzed by nitrogenase at ambient conditions through multiple proton and electron transfer steps, requiring a significant energy input delivered by adenosine triphosphate (ATP)^11,19–21^. Encouragingly, electrochemical N_2_ reduction using protons and electrons can be powered by renewable energy from solar or wind sources, offering a promising environmentally benign process for sustainable artificial N_2_ fixation at room temperature and pressure^22,23^. This process, however, is severely challenged by N_2_ activation and demands efficient catalysts for the N_2_ reduction reaction (NRR)^24–26^. NRR catalysts based on noble metals (e.g., Au^27,28^, Ru^29^, Rh^30^) show favorable activity, but widespread use is hindered by scarcity and high cost. Much attention has thus focused on designing and developing non-noble-metal alternatives but with low Faradaic efficiency (FE), including Fe_2_O_3_-CNT^31^, Fe_3_O_4_^32^, Li^+^-incorporated PEBCD/C^33^, MoS_2_^34^, (110)-oriented Mo nanofilm^35^, MoO_3_^36^, Mo_2_N^37^, etc. Recently, Lv et al. have reported improved NRR catalytic performance in an amorphous Bi_4_V_2_O_11_-crystalline CeO_2_ hybrid (BVC-A) with a high FE of 10.16% and a NH_3_ yield that can reach up to 23.21 μg h^–1^ mg^–1^_cat._ (ref. ^38^). Compared with the catalysts above, metal-free materials offer an obvious advantage of avoiding metal ion release, thereby reducing the environmental impact. N-doped nanocarbon was recently reported for N_2_ reduction electrocatalysis with a remarkable NH_3_ yield of 23.8 μg h^–1^ mg^–1^_cat._, but its FE is only 1.42%^39^. In this context, the idenfication of new metal-free NRR nanocatalysts that simultaneously achieve high NH_3_ formation rate and FE is highly desired, which, however, still remains a key challenge.

Boron carbide (B_4_C), one of the hardest materials in nature next to diamond and cubic boron nitride, possesses high mechanical strength, (electro)chemical stability, and good electronic conductivity, and much attention has focused on its electrochemical uses as electrode material or catalyst substrate for batteries and fuel cells^40–43^. Here we report our recent finding that B_4_C nanosheet behaves as a superb metal-free electrocatalyst toward artificial N_2_ fixation with excellent selectivity for NH_3_ formation under ambient conditions. In 0.1 M hydrochloric acid (HCl), it is capable of achieving an average NH_3_ formation rate and a FE as high as 26.57 μg h^–1^ mg^–1^_cat._ and 15.95% at –0.75 V, respectively, placing it among the most active aqueous-based NRR electrocatalysts. Notably, it also shows high electrochemical stability. In 0.1 M sodium sulfate (Na_2_SO_4_), the catalyst still exhibits good activity and selectivity with an NH_3_ yield of 14.70 μg h^–1^ mg^–1^_cat._ and a FE of 9.24%. Density functional theory (DFT) calculations suggest that the *NH_2_–*NH_2_→*NH_2_–*NH_3_ reaction is the rate-limiting step.

## Results

### Synthesis and characterization of boron carbide nanosheet

**Fig. 1:**
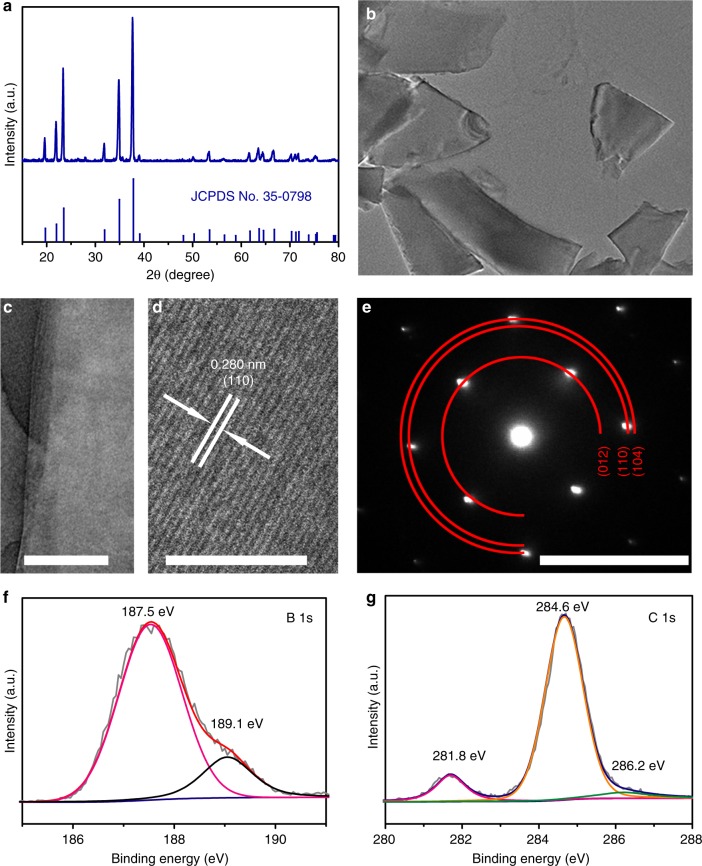
Structure, morphology, and composition characterizations. **a** X-ray diffraction (XRD) pattern for B_4_C. **b**, **c** Transmisson electron microscopy (TEM) micrograph (**b**) and further magnified TEM images (**c**) for B_4_C nanosheets. **d**, **e** High-resolution TEM image (**d**) and selected area electron diffraction (SAED) pattern for one B_4_C nanosheet (**e**). Scale bars, **b** 1 μm; **c** 300 nm; **d** 5 nm; **e** 5 nm^−1^. **f**, **g** X-ray photoelectric spectra of B_4_C nanosheets in the B 1*s* (**f**) and C 1*s* (**g**) regions

The B_4_C nanosheet was produced by liquid exfoliation of bulk B_4_C (see Methods for preparation details). As shown in Fig. 1a, the X-ray diffraction (XRD) pattern for B_4_C is highly crystalline with diffraction peaks at 19.7°, 22.0°, 23.5°, 31.9°, 34.9°, 37.8°, 53.5°, 63.7°, and 66.7° that are indexed to the (101), (003), (012), (110), (104), (021), (205), (125), and (220) planes of B_4_C phase (JCPDS No. 35-0798)^44^, respectively. Further characterization by scanning electron microscopy (SEM) and transmission electron microscopy (TEM) confirm the formation of nanosheets after liquid exfoliation, as shown in Supplementary Fig. 1 and Fig. 1b. The higher magnification TEM image (Fig. 1c) reveals the formation of few-layered B_4_C nanosheet. The high-resolution TEM (HRTEM) image (Fig. 1d) of such a nanosheet shows well-resolved lattice fringes with an interplanar distance of 0.280 nm indexed to the (110) plane of B_4_C. The corresponding selected area electron diffraction (SAED) pattern (Fig. 1e) shows well-defined rings indexed to the (012), (110), and (104) planes of B_4_C. X-ray photoelectron spectroscopy (XPS) spectra of B_4_C in C 1*s* (Fig. 1f) and B 1*s* (Fig. 1g) regions are in good agreement with reported results^45^. The peaks in B 1*s* are 187.5 eV and 189.1 eV, which can be associated with the B atoms in B−B and B−C bonds, respectively. The peaks of C 1*s* spectrum are 284.6 eV (C−C bond), 286.2 eV (C−O bond) and 281.8 eV (C−B bond). The Raman spectroscopy of the B_4_C presents characteristic Raman peaks at 270, 320, 481, 531, 728, 830, 1000, and 1088 cm^–1^ assigned to crystalline B_4_C (Supplementary Fig. 2)^46^.

### Electrocatalytic nitrogen reduction performance

**Fig. 2:**
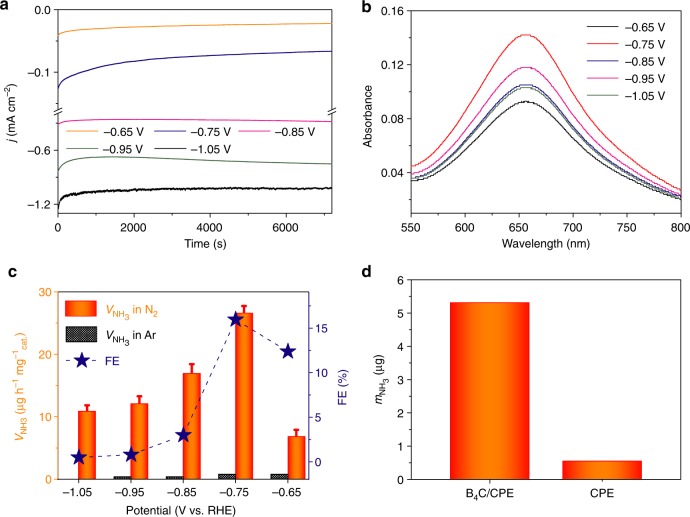
Electrocatalytic nitrogen reduction performance. **a** Chrono-amperometry curves at various potentials in N_2_-saturated 0.1 M HCl. **b** Ultraviolet-visible (UV-Vis) absorption spectra of the 0.1 M HCl electrolytes stained with indophenol indicator after electrolysis at a series of potentials for 2 h (7200 s). **c** NH_3_ yields and Faradaic efficiencies (FEs) at each given potential in 0.1 M HCl. **d** Amounts of NH_3_ generated with a carbon paper electrode (CPE) and a B_4_C/CPE electrode after 2-h electrolysis at potential of −0.75 V under ambient conditions

The NRR catalytic performance is examined at controllable applied voltages using a three-electrode system comprising of a graphite rod as a counter electrode, Ag/AgCl as a reference electrode and a B_4_C nanosheet-loaded carbon paper electrode (CPE) as a working electrode (B_4_C/CPE; B_4_C nanosheet loading: 0.1 mg cm^−2^). During electrolysis, N_2_ gas is bubbled into the cathode, where protons transported from the electrolyte (0.1 M HCl aqueous solution) can react with N_2_ on the surface of the catalyst to produce NH_3_. As shown in Fig. 2a, the current of chrono-amperometry curves at different potentials exert good stability. The current density starts high and then decreases to a steady state, which might be ascribed to double layer charging and a result of decreasing local concentration of H^+^ and N_2_ near the electrode surface^47^.

To confirm the successful N_2_ electroreduction in 0.1 M HCl, the production of both NH_3_ and a possible by-product hydrazine (N_2_H_4_) are spectrophotometrically evaluated after 2-h electrolysis operation by the indophenol blue method^7^ and the method of Watt and Chrisp^48^, respectively (the corresponding calibration curves are shown in Supplementary Fig. 3–4). Figure 2b shows the UV-Vis absorption spectra of electrolyte colored with indophenol indicator at a series of potentials under N_2_ bubbling. This detection of NH_3_ is concrete and unambiguous proof of NH_3_ formation via the electroreduction of N_2_ in our B_4_C/CPE platform at potential ranges from −0.65 V to −1.05 V.

The average NH_3_ yields and corresponding FEs were determined to exemplify the B_4_C nanosheet as an efficient catalyst for the fixation of inert N_2_ molecules into highly valuable NH_3_ (Fig. 2c). As observed, NH_3_ yields increase with more negative potential until reaching −0.75 V, where the maximum value of NH_3_ yield is calculated as 26.57 μg h^–1^ mg^–1^_cat._ with a FE of 15.95%, outperforming most reported aqueous-based NRR catalysts at ambient conditions (Supplementary Table 1). The NH_3_ yield and FE of NRR increase initially (−0.65 V to −0.75 V) and then start to decrease as the potential is negatively shifted to −1.05 V, which is attributed to the FE of the hydrogen evolution reaction that rises slowly (−0.65 to −0.75 V) and then rises rapidly beyond the cathodic polarization potential of −0.75 V (Supplementary Fig. 5)^49^. Of note, N_2_H_4_ was not detected, indicating this catalyst possesses excellent selectivity for NH_3_ formation (Supplementary Fig. 6). It is also important to mention that bare CPE has poor electrocatalytic NRR activity (Fig. 2d), revealing that the B_4_C nanosheet is highly active to catalyze N_2_ electroreduction.

To verify that the detected NH_3_ molecules mainly originate from the electrocatalyzed conversion of N_2_ by B_4_C/CPE, control experiments were carried out with an Ar-saturated electrolyte as a function of applied potential and with no potential applied to the electrodes under N_2_ gas (open circuit voltage). The corresponding UV-Vis absorption spectra (Supplementary Fig. 7) and calculated NH_3_ yields (Fig. 2c) show the presence of a tiny amount of NH_3_ that may come from sources of contamination (e.g., laboratory, equipment, membrane). A ^15^N isotopic labeling experiment was also performed to verify the N source of the produced NH_3_. As shown in Supplementary Fig. 8, the standard samples show a triplet coupling for ^14^NH_4_^+^ and a doublet coupling for ^15^NH_4_^+^ in the ^1^H nuclear magnetic resonance (^1^H NMR) spectra, and the use of ^14^N_2_ and ^15^N_2_ as the feeding gas yields ^14^NH_4_^+^ and ^15^NH_4_^+^, repsectively. These results provide another piece of evidence to strongly support that NH_3_ was produced by B_4_C-catalyzed electroreduction of N_2_.

**Fig. 3:**
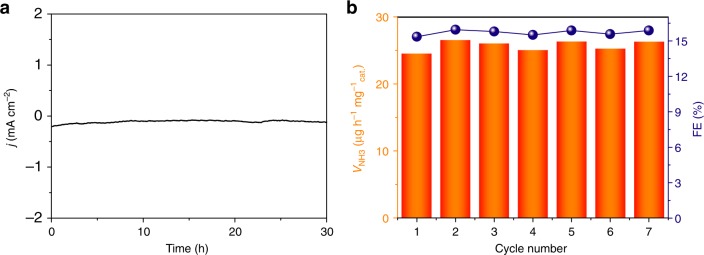
Durability tests. **a** Time-dependent current density curve and (**b**) recycling test of B_4_C/carbon paper electrode (CPE) at a potential of −0.75 V under ambient conditions

Stability is also a critical parameter of NRR performance for practical applications. Under sustained N_2_ gas flow, 30-h electrolysis at a potential of −0.75 V only leads to a slight decrease in current density (Fig. 3a). After long-term electrolysis, the NH_3_ yield for B_4_C/CPE shows only 8% decrease compared with the initial one (Supplementary Fig. 9). Furthermore, B_4_C/CPE presents small changes in NH_3_ yield and FE during consecutive recycling tests at −0.75 V for 7 times (Fig. 3b), indicating that high electrocatalytic activity for NRR is maintained very well. The TEM image of a B_4_C nanosheet after long-term electrocatalysis (Supplementary Fig. 10) shows almost no obvious change in morphology. XRD analysis (Supplementary Fig. 11) and XPS spectra (Supplementary Fig. 12) confirm that this catalyst is still B_4_C in nature after NRR. All these results indicate that this catalyst is robust enough to afford NRR electrocatalysis, which may be attributed to the excellent chemical and electrochemical stability of B_4_C in acid and its intrinsic high mechanical strength^42^.

The NRR activity of B_4_C/CPE was also assessed in neutral media (0.1 M Na_2_SO_4_). Production of NH_3_ and the possible by-product (N_2_H_4_) were evaluated by a spectrophotometry method^31,48^. The calibration curves are shown in Supplementary Fig. 13–14. B_4_C/CPE still exhibits excellent selectivity without N_2_H_4_ production (Supplementary Fig. 15). As shown in Supplementary Fig. 16–17, the NH_3_ yield can reach the highest value of 14.70 μg h^–1^ mg^–1^_cat._ with a FE of 9.24% at potential of −0.75 V. Time-dependent current density curves of B_4_C/CPE for NRR at different potentials suggest excellent stability (Supplementary Fig. 18).

## Discussion

To identify the active site and atomistic electrocatalytic processes of the NRR on the B_4_C surface, we used the exchange-correlation functional of Perdew, Burke, and Ernzerhof and the dispersion correction method of Grimme (PBE-D) in the framework of DFT to simulate the corresponding electrocatalytic reactions on the B_4_C (110) surface using a periodic slab model (see Methods for details). It is well known that the N_2_ adsorption on the catalyst surface is the first step to initialize the NRR and its initial adsorption configuration plays a vital role for subsequent catalytic reactions. Thus, we have first examined the N_2_ adsorption on the B_4_C (110) surface.

There are two main configurations available for the N_2_ adsorption on the B_4_C (110) surface. In the end-on configuration, only one terminal N atom is bonded to the B atom on the B_4_C (110) surface; in the side-on configuration, two terminal N atoms are separately bonded to two vertical B atoms that are located on two adjacent boron clusters (Supplementary Fig. 19). The N_2_ adsorption potential energies in these two structures are calculated to be 0.65 eV and 0.63 eV at the PBE-D level (free energies: 0.41 eV and 0.34 eV, Supplementary Table 2). Since both configurations have similar energy profiles for the electrochemical N_2_ fixation reaction on the B_4_C (110) surface, in the following, we have merely focused on discussing the one starting from the end-on configuration.

**Fig. 4:**
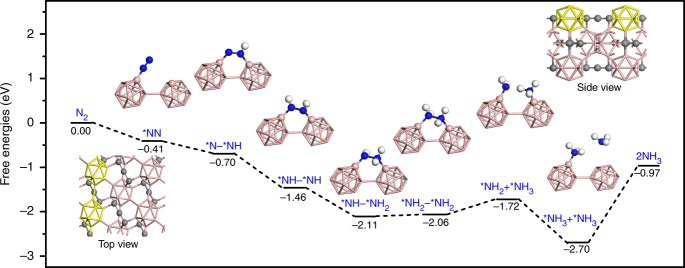
Density functional theory calculations. Density functional theory (DFT) of Perdew, Burke, and Ernzerhof with the dispersion correction method of Grimme (PBE-D) calculated energy profiles for the electrocatalytic N_2_ fixation reaction on the B_4_C (110) surface starting from the end-on adsorption structure (see optimized structures in Supplementary Fig. 20; energy profiles from the side-on adsorption structure in Supplementary Fig. 22). Color code: blue, N; rose, B; gray, C; white, H; the asterisk * denotes an adsorption site

Figure 4 shows our DFT computed energy profiles for the electrocatalytic NRR processes on the B_4_C (110) surface starting from the end-on adsorption structure (optimized structures, Supplementary Fig. 20). The initial adsorption of molecular nitrogen in this end-on configuration releases 0.41 eV free energy. It can be found that the reaction process from the adsorbed *NN to *NH–*NH_2_ species is completely barrierless and releases 1.70 eV free energy at potential of 0.00 V. The following reaction step, i.e., *NH–*NH_2_ → *NH_2_–*NH_2_, is also facile because of a small energy gap of 0.05 eV (PBE-D level). Different from the situation of other electrocatalyts, the rate-limiting step of the NRR on the B_4_C (110) surface corresponds to the *NH_2_–*NH_2_ → *NH_2_ + *NH_3_ reaction, which needs to overcome a barrier of 0.34 eV at potential of 0.00 V (i.e., from −2.06 to −1.72 eV). The final electrocatalytic addition reaction of a proton and electron pair on the adsorbed *NH_2_ species is barrierless. By contrast, the desorption of the NH_3_ molecule, i.e. *NH_3_→NH_3_, demands 1.73 eV free energy. Nevertheless, such a process remains energetically efficient concerning a lot of accumulated free energy in previous reaction processes (2.70 eV at potential of 0.00 V; see Fig. 4). Further discussion on the limiting potential of the NRR and the associated energy profile (Supplementary Fig. 21) can be found in Supplementary information. Finally, the energy profiles for the electrocatalytic processes starting from the side-on adsorption structure are similar to those from the end-on one (Supplementary Fig. 22).

In summary, a B_4_C nanosheet has been experimentally proven as a superior metal-free electrocatalyst for artificial N_2_ fixation to NH_3_ with excellent selectivity at room temperature and ambient pressure. This catalyst achieves a high NH_3_ yield of 26.57 µg h^–1^ mg^–1^_cat._ and a high FE of 15.95% at a potential of −0.75 V in 0.1 M HCl, with high electrochemical stability. Impressively, B_4_C/CPE still exhibits good NRR activity in neutral media. Further DFT calculations reveal that the *NH_2_–*NH_2_→*NH_2_–*NH_3_ reaction is the rate-limiting step. This study not only provides an attractive metal-free electrocatalyst material for NH_3_ synthesis, but also opens up an exciting new avenue to the rational design of B_4_C-based nanocatalysts with enhanced performance for N_2_-fixation applications.

## Methods

### Sample preparation

Commercial bulk B_4_C was purchased from Aladdin Ltd. (Shanghai, China). All reagents were analytical reagent grade without further purification. The water used throughout all experiments was purified through a Millipore system. A total of 1 g bulk B_4_C was dispersed in 10 mL ethanol and stripped by ultrasonic cell disruptor for 1 h. Subsequently, the resulting dispersion was centrifuged for 10 min at 3000 rpm and the supernatant containing B_4_C nanosheet was decanted gently. Next, 710 µL of the obtained solution was added into 250 µL H_2_O containing 40 µL of 5 wt% Nafion and sonicated for 1 h to form a homogeneous ink. Then, 50 µL of the dispersion was loaded onto a carbon paper electrode with area of 1 × 1 cm^2^ and dried under ambient conditions, the catalyst loading mass is 0.1 mg cm^–2^.

### Characterization

XRD pattern was recorded using a LabX XRD-6100 X-ray diffractometer, with a Cu Kα radiation (40 kV, 30 mA) of wavelength 0.154 nm (SHIMADZU, Japan). The structures of the samples were determined by TEM images on a HITACHI H-8100 electron microscopy (Hitachi, Tokyo, Japan) operated at 200 kV. SEM image was obtained on a Hitachi S-4800 field emission scanning electron microscope at an accelerating voltage of 20 kV. XPS measurements were performed on an ESCALABMK II X-ray photoelectron spectrometer using Mg as the exciting source. The absorbance data of spectrophotometer were measured on SHIMADZU UV-1800 UV-Vis spectrophotometer. A gas chromatograph (SHIMADZU, GC-2014C) equipped with MolSieve 5A column and Ar carrier gas was used for H_2_ quantifications. Gas-phase product was sampled every 1000 s using a gas-tight syringe (Hamilton). ^1^H NMR spectra were collected on a superconducting-magnet NMR spectrometer (Bruker AVANCE III HD 500 MHz) and dimethyl sulphoxide was used as an internal to calibrate the chemical shifts in the spectra.

### Electrocatalytic nitrogen reduction measurements

The reduction of N_2_ gas (99.99%) was carried out in a two-compartment cell under ambient condition, which was separated by Nafion 211 membrane. The membrane was protonated by first boiling in ultrapure water for 1 h and treating in H_2_O_2_ (5%) aqueous solution at 80 °C for another 1 h, respectively. And then, the membrane was treaded in 0.5 M H_2_SO_4_ for 3 h at 80 °C and finally in water for 6 h. The electrochemical experiments were carried out with an electrochemical workstation (CHI 660E). The potentials reported in this work were converted to reversible hydrogen electrode (RHE) scale via calibration with the following equation: E (vs. RHE) = E (vs. Ag/AgCl) + 0.256 V and the presented current density was normalized to the geometric surface area. For electrochemical N_2_ reduction, chrono-amperometry tests were conducted in N_2_-saturated 0.1 M HCl solution (the HCl electrolyte was purged with N_2_ for 30 min before the measurement).

### Quantification of ammonia

When tested in 0.1 M HCl, the concentration of NH_3_ produced was spectrophotometrically determined by the indophenol blue method^7^. Typically, 2 mL HCl electrolyte was taken from the cathodic chamber, and then 2 mL of 1 M NaOH solution containing 5% salicylic acid and 5% sodium citrate was added into this solution. Subsequently, 1 mL of 0.05 M NaClO and 0.2 mL of 1% C_5_FeN_6_Na_2_O·2H_2_O were add into the above solution. After standing at room temperature for 2 h, the UV-Vis absorption spectrum was measured at a wavelength of 655 nm. The concentration-absorbance curves were calibrated using standard NH_3_ solution (Supplementary Fig. 3) with a series of concentrations. The fitting curve (*y* = 1.130*x* + 0.078, *R*^2^ = 0.999) shows good linear relation of absorbance value with NH_3_ concentration by three times independent calibrations. When tested in 0.1 M Na_2_SO_4_, the NH_3_ concentration was measured by a spectrophotometry method^31^. In detail, 4 mL of post-tested solution was removed from the cathodic chamber. Then, 50 μL of oxidizing solution (NaClO (*ρ*_Cl_ = ~4–4.9) and 0.75 M NaOH), 500 µL of coloring solution (0.4 M C_7_H_5_O_3_Na and 0.32 M NaOH) and 50 µL of catalyst solution (0.1 g Na_2_[Fe(CN)_5_NO] 2H_2_O diluted to 10 mL with deionized water) were added sequentially to the sample solution. After standing at 25 °C for 1 h, the UV-Vis absorption spectra were performed. The concentration of indophenol blue was determined using the absorbance at a wavelength of 655 nm. The concentration-absorbance curve was calibrated using standard NH_3_ solution with a series of concentrations. The fitting curve (*y* = 0.753*x* + 0.026, *R*^2^ = 0.999) shows good linear relation of absorbance value with NH_3_ concentration by three times independent calibrations (Supplementary Fig. 13).

### Quantification of hydrazine

The amount of N_2_H_4_ present in the electrolyte was estimated by the method of Watt and Chrisp^48^. A mixed solution of 5.99 g C_9_H_11_NO, 30 mL HCl, and 300 mL ethanol was used as a color reagent. Calibration curve was plotted as follow: first, preparing a series of standard solutions; second, adding 5 mL above prepared color reagent and stirring 20 min at room temperature; finally, the absorbance of the resulting solution was measured at 455 nm, and the yields of N_2_H_4_ were estimated from a standard curve using 5 mL residual electrolyte and 5 mL color reagent. Absolute calibration of this method was achieved using N_2_H_4_ solutions of known concentration as standards, and the fitting curve shows good linear relation of absorbance with N_2_H_4_ concentration in 0.1 M HCl (Supplementary Fig. 4, *y* = 0.545*x* + 0.031, *R*^2^ = 0.999) and 0.1 M N_2_SO_4_ (Supplementary Fig. 14, *y* = 1.199*x* + 0.051, *R*^2^ = 0.999) by three times independent calibrations.

### Calculation of the Faradaic efficiency and yield

The FE for N_2_ reduction was defined as the amount of electric charge used for synthesizing NH_3_ divided the total charge passed through the electrodes during the electrolysis. The total amount of NH_3_ produced was measured using colorimetric methods. Assuming three electrons were needed to produce one NH_3_ molecule, the FE could be calculated as follows:

1 $${\mathrm{FE}} = \frac{{{\mathrm{3 \times F \times C}}_{{\mathrm{NH}}_3}{\mathrm{ \times V}}}}{{{\mathrm{17 \times Q}}}}.$$

The rate of NH_3_ formation $({v_{{\mathrm{NH}}_3}})$ was calculated using the following equation:

2 $$v_{{\mathrm{NH}}_3} = \frac{{{\mathrm{C}}_{{\mathrm{NH}}_3}{\mathrm{ \times V}}}}{{{t \times }m_{{\mathrm{cat}}{\mathrm{.}}}}}.$$

where *F* is the Faraday constant, ${\rm C}_{{\rm NH}_3}$ is the measured NH_3_ concentration, *V* is the volume of the HCl electrolyte for NH_3_ collection, *t* is the reduction time and *m*_cat_. is the catalyst loading mass.

FE for H_2_ was calculated according to following equation:

3 $${\mathrm{FE}} = 2 \times {\mathrm{F}} \times {n}/{\mathrm{Q}},$$

where F is the Faraday constant; *n* is the actually produced H_2_ (mol), and Q is the quantity of applied electricity.

### Calculation details

All DFT calculations are performed using the DMol^3^ module implemented in the Material Studio 8.0 package^50,51^. The generalized gradient approximation (GGA) with the PBE exchange-correlation functional is employed^52^. The empirical dispersion correction proposed by Grimme is used to consider weak van der Waals interaction^53^. The build-in double numerical plus polarization (DNP) basis set is used to expand the electronic wavefunction. A Monkhorst-Pack k-point grids of 2 × 1 × 1 are used (see Supplementary Table 3 for benchmark). Self-consistent field (SCF) calculations are performed with a convergence criterion of 10^−6^ au on the total energy and electronic computations. Since the bulk water layer only slightly stabilizes the NRR intermediates^54^, we have therefore adopted the conductor-like screening model (COSMO) to simulate solvent effects^55^.

A three-layer 1 × 2 periodic slab model is used to represent the B_4_C (110) surface that is observed in our HRTEM image (see Supplementary Table 4 for benchmark). A 15 Å vacuum layer is used between the two neighboring slabs to avoid artificial interaction. In geometric optimizations, all atoms except those in the bottom layer are fully relaxed. The adsorption energy (E_ads_) of species on the B_4_C (110) surface is defined as:

4 $${\mathrm{E}}_{{\mathrm{ads}}} = {\mathrm{E}}_{{\mathrm{slab}}} + {\mathrm{E}}_{{\mathrm{mol}}} - {\mathrm{E}}_{{\mathrm{slab}}/{\mathrm{mol}}},$$

where E_slab_ and E_mol_ are the energies of the isolated slab and species, respectively; and E_slab/mol_ is the energy of the species-adsorbed slab system. The NRR process includes six net coupled proton and electron transfer (CPET) steps (N_2_ + 6H^+^ + 6e^−^ → 2NH_3_). Based on previous theoretical studies^54^, gaseous hydrogen is used as the source of protons to simulate the reaction at the anode, i.e., H_2_ ↔ 2H^+^ + 2e^−^. Each CPET step includes the transfer of a proton coupled with an electron from solution to an adsorbed species on the B_4_C (110) surface. For each fundamental step, the Gibbs free energy change (ΔG) is calculated based on the standard hydrogen electrode (SHE) model proposed by Nørskov et al.^56–58^ in which the chemical potential of a proton-coupled-electron pair i.e., µ(H^+^) + µ(e^−^) is equal to half of the chemical potential of gaseous hydrogen i.e., 1/2 µ(H_2_) at a potential of 0 V. Accordingly, the ΔG value can be obtained as follows:

5 $${\mathrm{\Delta G}} = {\mathrm{\Delta E}} + {\mathrm{\Delta ZPE}} - {\mathrm{T\Delta S}} + {\mathrm{\Delta G}}_{\mathrm{U}} + {\mathrm{\Delta G}}_{{\mathrm{pH}}},$$

where ΔE is the electronic energy difference, ΔZPE is the change in zero-point energies, *T* is the temperature (*T* = 298.15 K), and ΔS is the change of entropy. ΔG_U_ is the free energy contribution connected to electrode potential U. ΔG_pH_ is the H^+^ free energy correction by the concentration. It is calculated through ΔG_pH_ = 2.303 × *k*_B_T × pH where *k*_B_ is the Boltzmann constant and the value of pH is assumed to be zero in this work. It can be found that the free energy change of each elementary step is increased as the pH value increases. The zero-point energies and entropies of the NRR species are determined from the vibrational frequencies in which only the adsorbed species’ vibrational modes are computed explicitly and the B_4_C (110) surface is fixed. The entropies and vibrational frequencies of molecules in the gas phase are taken from the NIST database. [http://cccbdb.nist.gov/]

### Data availability

The data described in this paper are available from the authors upon reasonable request.

## Electronic supplementary material

Supplementary Information

## References

[1] Smil V (1999). Detonator of the population explosion. Nature.

[2] Schlögl R (2003). Catalytic synthesis of ammonia—a “never-ending story”?. Angew. Chem. Int. Ed..

[3] Rosca V, Duca M, DeGroot MT, Koper MTM (2009). Nitrogen cycle electrocatalysis. Chem. Rev..

[4] Vegge, T. et al. Indirect hydrogen storage in metal ammines. In: *Solid State Hydrogen Storage: Materials and Chemistry* (British Welding Research Association, Cambridge, UK, 2008).

[5] MacKay BA, Fryzuk MD (2004). Dinitrogen coordination chemistry: on the biomimetic borderlands. Chem. Rev..

[6] Kitano M (2012). Ammonia synthesis using a stable electride as an electron donor and reversible hydrogen store. Nat. Chem..

[7] Zhu D, Zhang L, Ruther RE, Hamers RJ (2013). Photo-illuminated diamond as a solid-state source of solvated electrons in water for nitrogen reduction. Nat. Mater..

[8] Shima T (2013). Dinitrogen cleavage and hydrogenation by a trinuclear titanium polyhydride complex. Science.

[9] MacLeod KC, Holland PL (2013). Recent developments in the homogeneous reduction of dinitrogen by molybdenum and iron. Nat. Chem..

[10] Čorić I, Mercado BQ, Bill E, Vinyard DJ, Holland PL (2015). Binding of dinitrogen to an iron-sulfur-carbon site. Nature.

[11] Brown KA (2016). Light-driven dinitrogen reduction catalyzed by a CdS: nitrogenase MoFe protein biohybrid. Science.

[12] Eizawa A (2017). Remarkable catalytic activity of dinitrogen-bridged dimolybdenum complexes bearing NHC-based PCP-pincer ligands toward nitrogen fixation. Nat. Commun..

[13] Légaré MA (2018). Nitrogen fixation andreduction at boron. Science.

[14] Smil V (1997). Global population and the nitrogen cycle. Sci. Am..

[15] Jennings JR (1991). Catalytic ammonia synthesis: fundamentals and practice..

[16] Aika, K. et al. *Ammonia, catalysis and manufacture*. (Springer-Verlag Berlin Heidelberg, New York, Dordrecht, 1995).

[17] Leigh, G. J. Haber–bosch and other industrial processes. In: Catalysts for Nitrogen Fixation. (Kluwer Academic Publishers, 2004).

[18] Nørskov, J., Chen, J., Miranda, R., Fitzsimmons, T. & Stack, R. *Sustainable ammonia synthesis*, DOE roundtable report. (U.S. department of energy Office of Science, Washington, DC, 2016).

[19] Tanaka H, Nishibayashi Y, Yoshizawa K (2016). Interplay between theory and experiment for ammonia synthesis catalyzed by transition metal complexes. Acc. Chem. Res..

[20] Chan MK, Kim J, Rees D (1993). The nitrogenase FeMo-cofactor and P-cluster pair: 2.2 Å resolution structures. Science.

[21] Burgess BK, Lowe DJ (1996). Mechanism of molybdenum nitrogenase. Chem. Rev..

[22] Shipman MA, Symes MD (2017). Recent progress towards the electrosynthesis of ammonia from sustainable resources. Catal. Today.

[23] Kyriakou V, Garagounis I, Vasileiou E, Vourros A, Stoukides M (2017). Progress in the electrochemical synthesis of ammonia. Catal. Today.

[24] van der Ham CJM, Koper MTM, Hetterscheid DGH (2014). Challenges in reduction of dinitrogen by proton and electron transfer. Chem. Soc. Rev..

[25] Seh ZW (2017). Combining theory and experiment in electrocatalysis: insights into materials design. Science.

[26] Guo C, Ran J, Vasileff A, Qiao SZ (2018). Rational design of electrocatalysts and photo(electro)catalysts for nitrogen reduction to ammonia (NH_3_) under ambient conditions. Energy Environ. Sci..

[27] Shi MM (2017). Au sub-nanoclusters on TiO_2_ toward highly efficient and selective electrocatalyst for N_2_ conversion to NH_3_ at ambient conditions. Adv. Mater..

[28] Bao D (2017). Electrochemical reduction of N_2_ under ambient conditions for artificial N_2_ fixation and renewable energy storage using N_2_/NH_3_ cycle. Adv. Mater..

[29] Kugler K, Luhn M, Schramm JA, Rahimi K, Wessling M (2015). Galvanic deposition of Rh and Ru on randomly structured Ti felts for the electrochemical NH_3_ synthesis. Phys. Chem. Chem. Phys..

[30] Liu Hm (2018). Surfactant-free atomically ultrathin rhodium nanosheets nanoassemblies for efficient nitrogen electroreduction. J. Mater. Chem. A.

[31] Chen S (2017). Electrocatalytic synthesis of ammonia at room temperature and atmospheric pressure from water and nitrogen on a carbon-nanotube-based electrocatalyst. Angew. Chem. Int. Ed..

[32] Liu, Q. et al. Ambient N_2_ fixation to NH_3_ electrocatalyzed by spinel Fe_3_O_4_ nanorod. *Nanoscale*, **10**, 14386–14389 (2018). 10.1039/c8nr04524k 30027985

[33] Chen GF (2017). Ammonia electrosynthesis with high selectivity under ambient conditions via a Li^+^ incorporation strategy. J. Am. Chem. Soc..

[34] Zhang L (2018). Electrochemical ammonia synthesis via nitrogen reduction reaction on MoS_2_ catalyst: theoretical and experimental studies. Adv. Mater..

[35] Yang D, Chen T, Wang Z (2017). Electrochemical reduction of aqueous nitrogen (N_2_) at a low overpotential on (110)-oriented Mo nanofilm. J. Mater. Chem. A.

[36] Han J (2018). MoO_3_ nanosheets for efficient electrocatalytic N_2_ fixation to NH_3_. J. Mater. Chem. A.

[37] Ren, X. et al. Electrochemical N_2_ fixation to NH_3_ under ambient conditions: Mo_2_N nanorod as a highly efficient and selective catalyst. *Chem. Commun*. **54**, 8474–8477 (2018). 10.1039/c8cc03627f 30003198

[38] Lv C (2018). An amorphous noble-metal-free electrocatalyst enables N_2_ fixation under ambient conditions. Angew. Chem. Int. Ed..

[39] Liu Y (2018). Facile ammonia synthesis from electrocatalytic N_2_ reduction under ambient conditions on N-doped porous carbon. ACS Catal..

[40] Minakshi M, Blackford MG (2010). Electrochemical characteristics of B_4_C or BN added MnO_2_ cathode material for alkaline batteries. Mater. Chem. Phys..

[41] Lv H, Peng T, Wu P, Pan M, Mu S (2012). Nano-boron carbide supported platinum catalysts with much enhanced methanol oxidation activity and CO tolerance. J. Mater. Chem..

[42] Mu S (2016). Nano-size boron carbide intercalated graphene as high performance catalyst supports and electrodes for PEM fuel cells. Carbon N. Y..

[43] Song S (2017). B_4_C as a stable non-carbon-based oxygen electrode material for lithiumoxygen batteries. Nano Energy.

[44] Kou Z, Guo B, He D, Zhang J, Mu S (2017). Transforming two-dimensional boron carbide into boron and chlorine dual-doped carbon nanotubes by chlorination for efficient oxygen reduction. ACS Energy Lett..

[45] Vineesh TV (2015). Bifunctional electrocatalytic activity of boron-doped graphene derived from boron carbide. Adv. Energy Mater..

[46] Reddy KM, Liu P, Hirata A, Fujita T, Chen MW (2013). Atomic structure of amorphous shear bands in boron carbide. Nat. Commun..

[47] Zhou F (2017). Electro-synthesis of ammonia from nitrogen at ambient temperature and pressure in ionic liquids. Energy Environ. Sci..

[48] Watt GW, Chrisp JD (1952). Spectrophotometric method for determination of hydrazine. Anal. Chem..

[49] Oshikiri T, Ueno K, Misawa H (2016). Selective dinitrogen conversion to ammonia using water and visible light through plasmon-induced charge separation. Angew. Chem. Int. Ed..

[50] Delley B (1990). An all-electron numerical method for solving the local density functional for polyatomic molecules. J. Chem. Phys..

[51] Delley B (2000). From molecules to solids with the DMol^3^ approach. J. Chem. Phys..

[52] Perdew JP, Burke K, Ernzerhof M (1996). Generalized gradient approximation made simple. Phys. Rev. Lett..

[53] Grimme S (2006). Semiempirical GGA-type density functional constructed with a long-range dispersion correction. J. Comput. Chem..

[54] Skúlason E (2012). A theoretical evaluation of possible transition metal electro-catalysts for N_2_ reduction. Phys. Chem. Chem. Phys..

[55] Klamt A, Schüürmann G (1993). COSMO: a new approach to dielectric screening in solvents with explicit expressions for the screening energy and its gradient. J. Chem. Soc., Perkin Trans. 2.

[56] Nørskov JK, Rossmeisl J, Logadottir A, Lindqvist L (2004). Origin of the overpotential for oxygen reduction at a fuel-cell cathode. J. Phys. Chem. B.

[57] Rossmeisl J, Logadottir A, Nørskov JK (2005). Electrolysis of water on (oxidized) metal surfaces. Chem. Phys..

[58] Peterson AA, Abild-Pedersen F, Studt F, Rossmeisl J, Nørskov JK (2010). How copper catalyzes the electroreduction of carbon dioxide into hydrocarbon fuels. Energy Environ. Sci..

