# Supplementary material for: High-performance artificial nitrogen fixation at ambient conditions using a metal-free electrocatalyst
Source: Nat Commun. 2018 Aug 28;9:3485. doi: 10.1038/s41467-018-05758-5 (PMC6113289; doi:10.1038/s41467-018-05758-5)
Supplement: Supplementary file 1 — Supplementary Information [file 41467_2018_5758_MOESM1_ESM.pdf]

Supplmentary Information

High-performance artificial nitrogen fixation at ambient conditions using a metal-free electrocatalyst

Qiu et al.

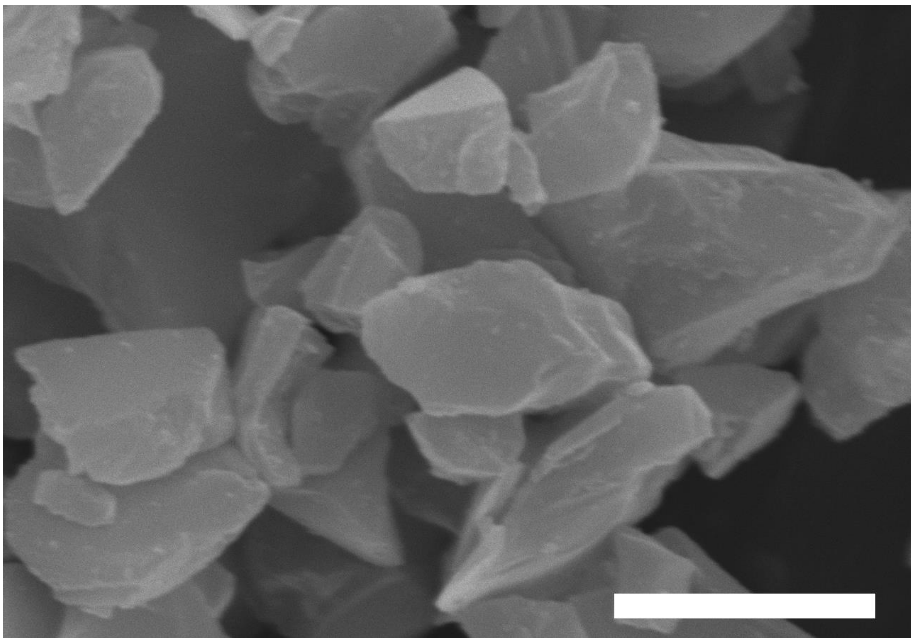

Supplementary Figure 1. SEM image of bulk B_4C. The scale bar is 2 μm.

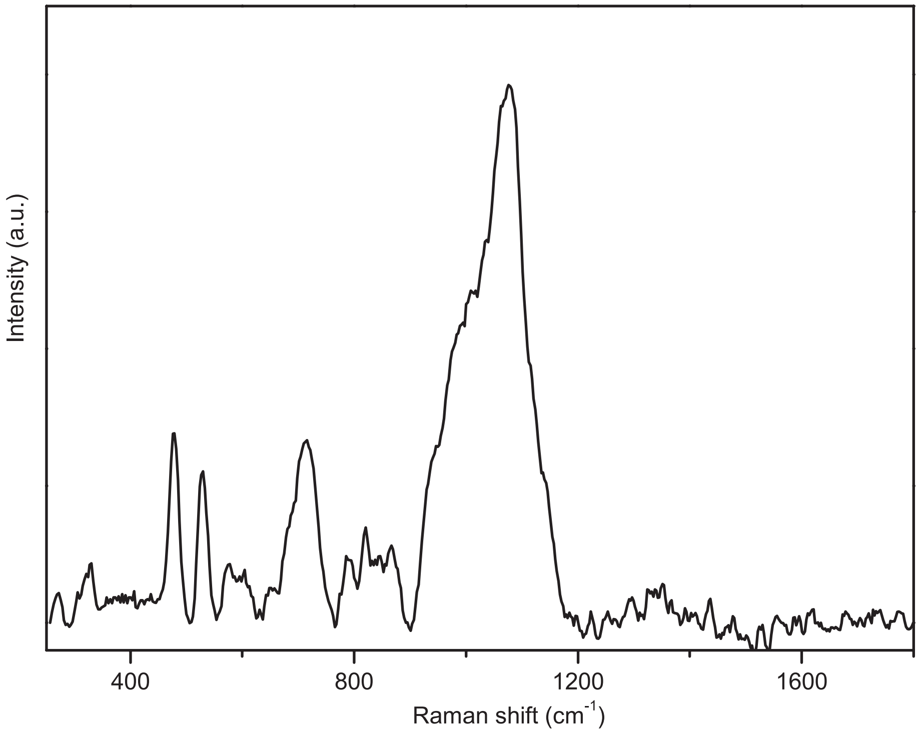

Supplementary Figure 2. Raman spectrum of B_4C.

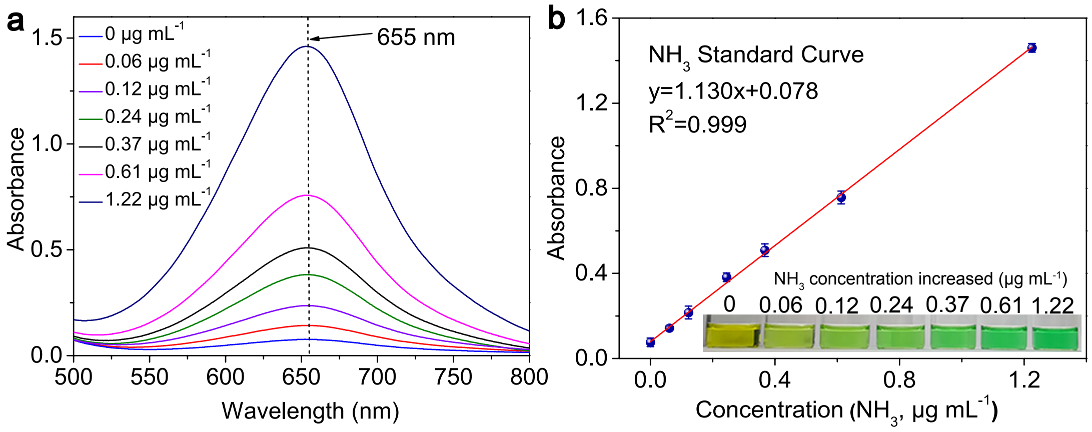

Supplementary Figure 3. (a) UV-Vis absorption spectra of indophenol assays with NH_3 after incubated for 2 h at room temperature. (b) Calibration curve used for estimation of NH_3.

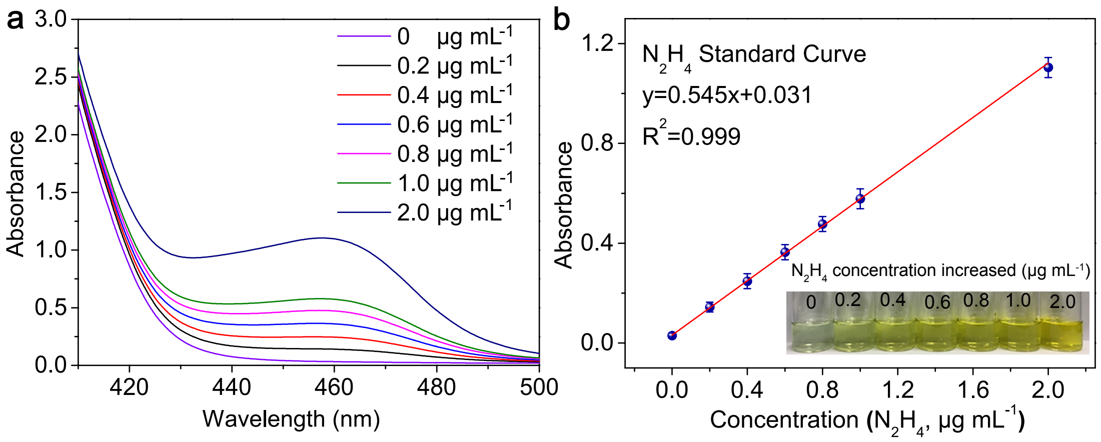

Supplementary Figure 4. (a) UV-Vis absorption spectra of various N_2H_4 concentration after incubated for 20 min at room temperature. (b) Calibration curve used for calculation of N_2H_4 concentration.

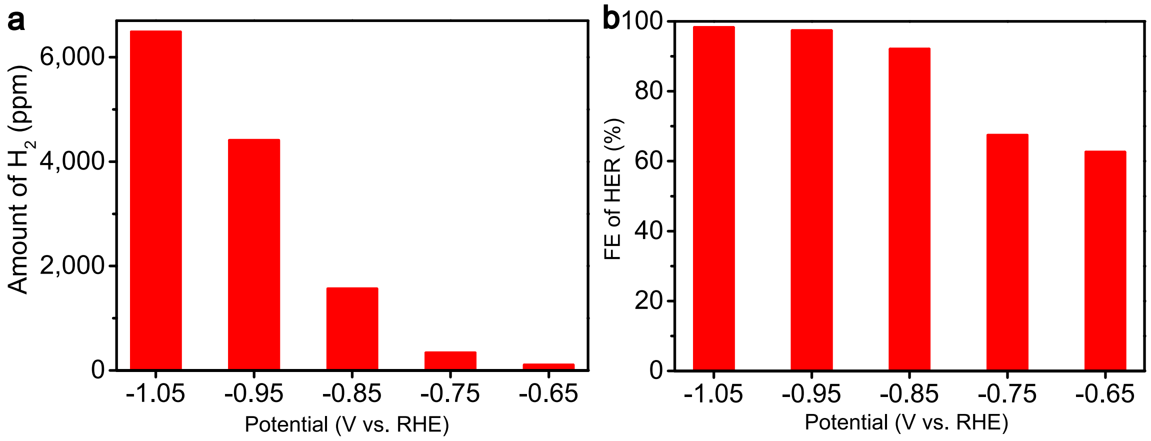

Supplementary Figure 5. (a) Amounts of H_2 from gas chromatography (GC) data of the gas from the headspace of the cell for the NRR on the B_4C/CPE catalyst in N_2-saturated 0.1 M HCl at various potentials. (b) The calculated FEs of HER. Combing the data with the obtained NH_3 selectivity, the unaccounted value may be attributed to the capacitance of the carbon support as well as dynamic hydrogen adsorption and absorption on B_4C.

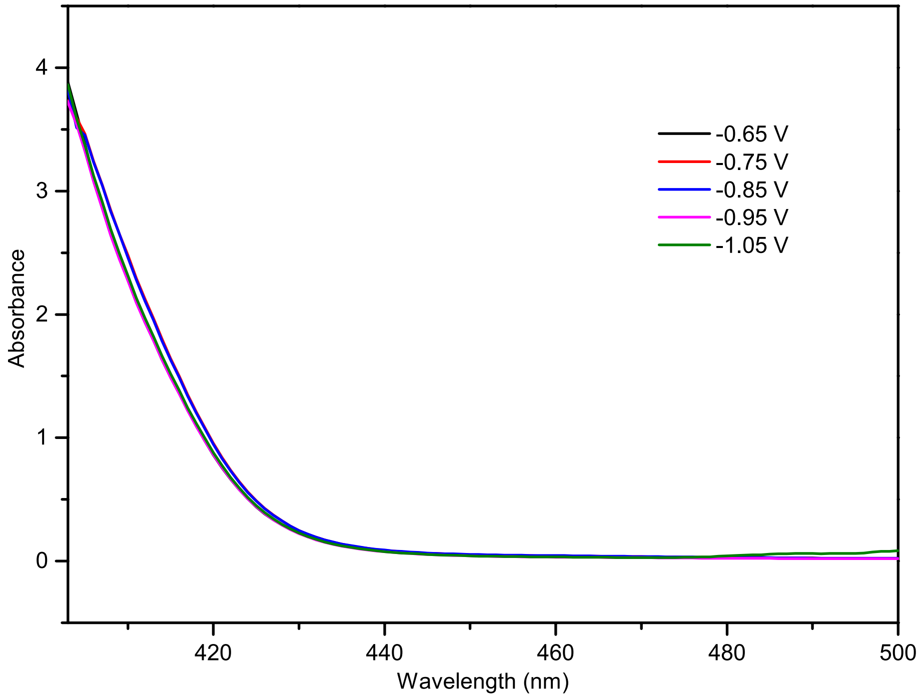

Supplementary Figure 6. UV-Vis absorption spectra of the 0.1 M HCl electrolytes estimated by the method of Watt and Chrisp after 2-h electrolysis in N_2 at each given potential under ambient conditions.

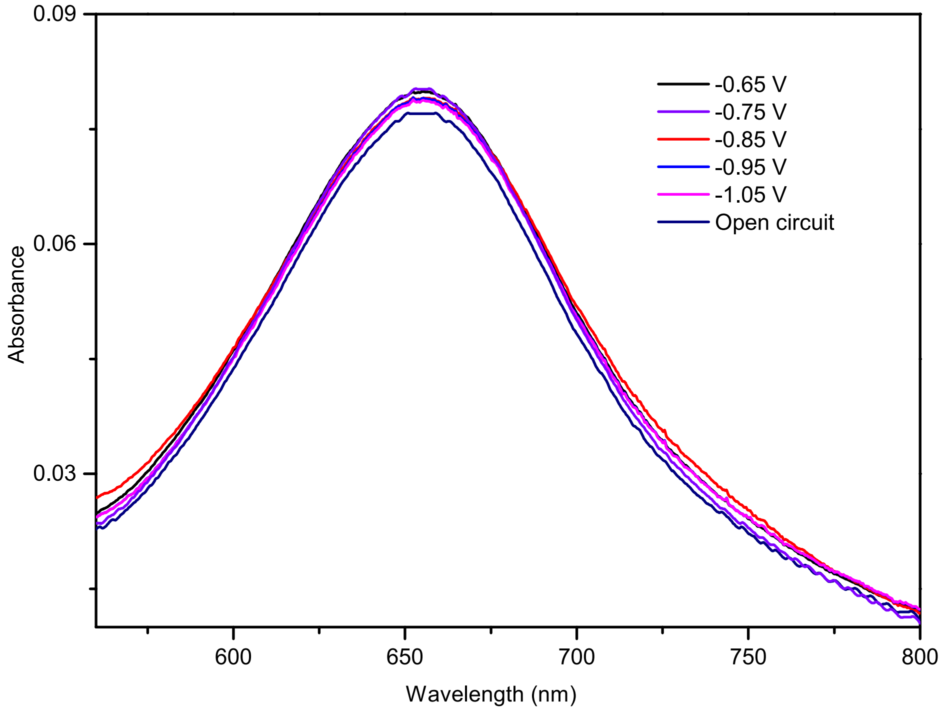

Supplementary Figure 7. UV-Vis absorption spectra of the 0.1 M HCl electrolytes stained with indophenol indicator after 2-h electrolysis under Ar at each given potential and N_2-saturated solution at open circuit potential.

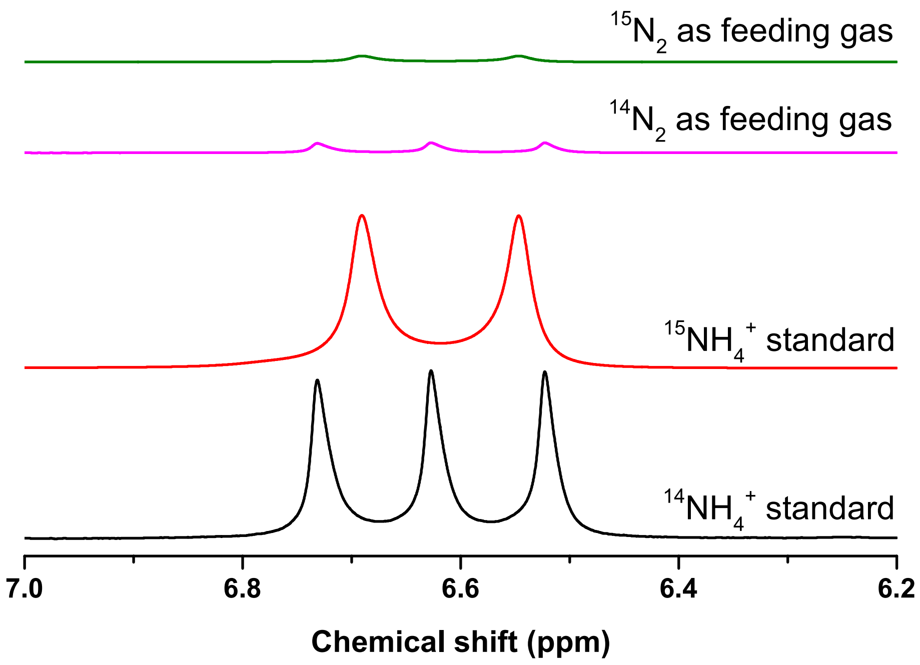

Supplementary Figure 8. ^{15}N isotope labeling experiment. ^{1}H NMR spectra for the post-electrolysis 0.1 M HCl electrolytes with $^{15}N_2$, $^{14}N_2$ as the feeding gas. Also shown are the spectra for $^{15}NH_4^+$ and $^{14}NH_4^+$ standard samples.

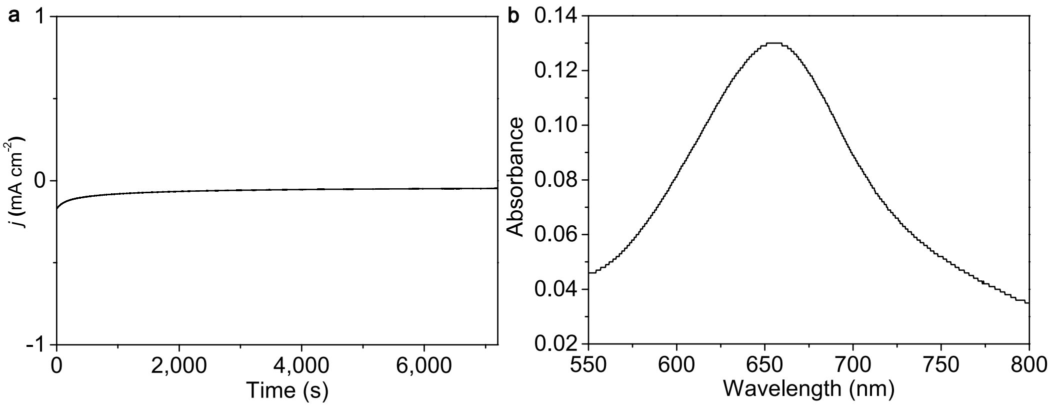

Supplementary Figure 9. (a) Chrono-amperometry cruve at –0.75 V in N_2-saturated 0.1 M HCl. (b) Corresponding UV-Vis absorption spectrum of the electrolyte stained with indophenol indicator after electrolysis.

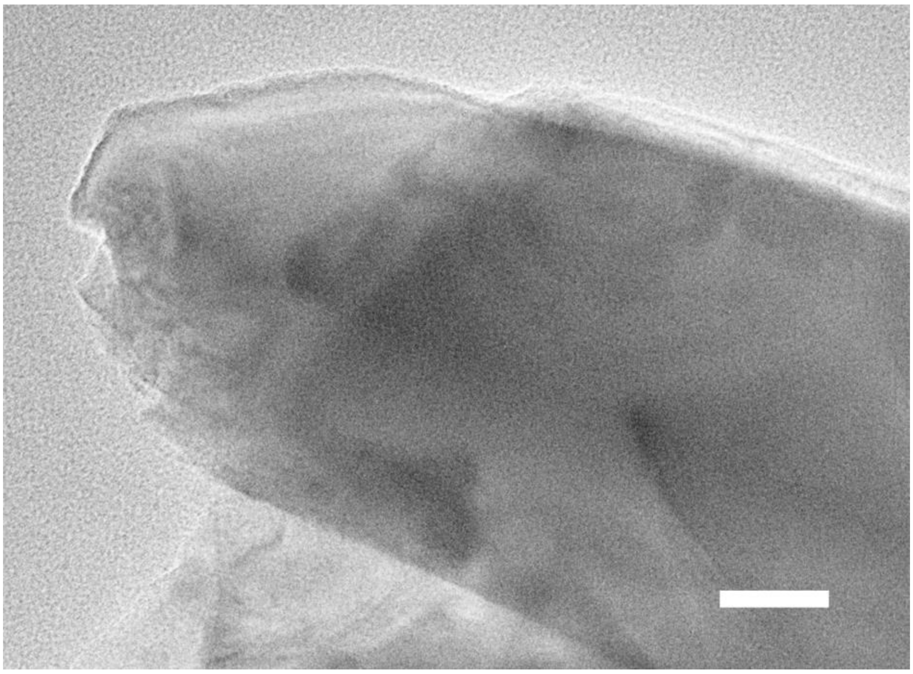

Supplementary Figure 10. TEM image of B_4C nanosheets after long-term stability test. The scale bar is 100 nm.

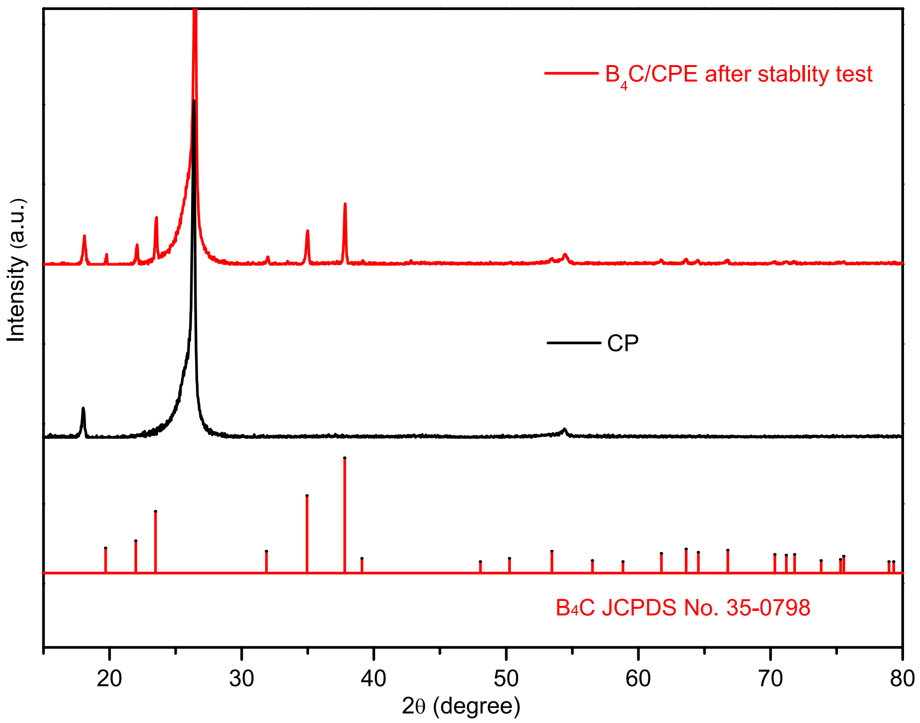

Supplementary Figure 11. XRD patterns of B_4C/CPE (red line) and CP (black line) after long-term stability test.

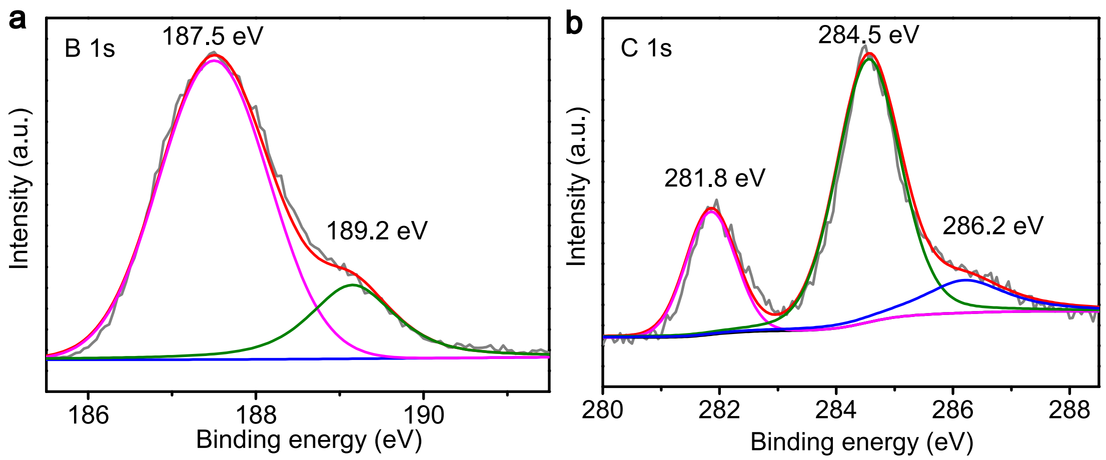

Supplementary Figure 12. XPS spectra of B_4C in the B 1s (a) and C 1s (b) regions after long-term stability test. The peak of 286.2 eV in B 1s increased after stability test, which is attributed to the increasing of adsorbed O.

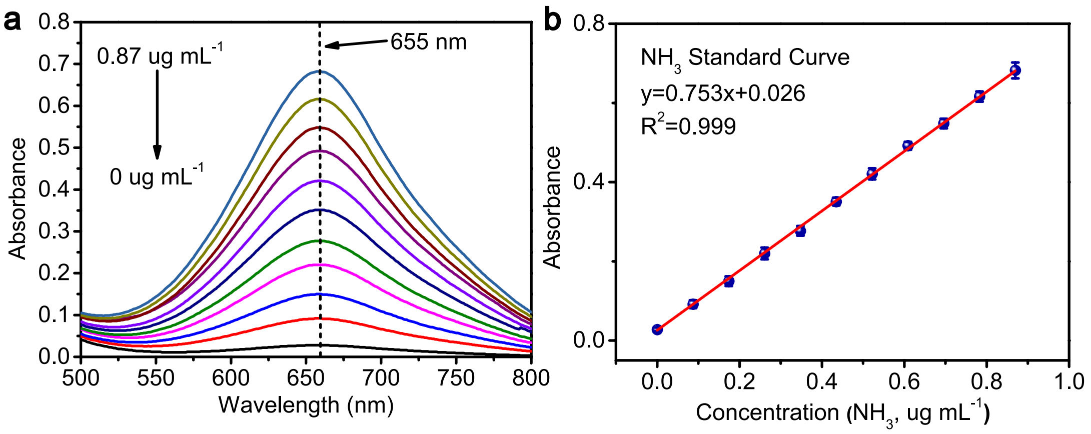

Supplementary Figure 13. (a) UV-Vis absorption spectra of indophenol assays with NH_3 after incubated for 1 h at room temperature. (b) Calibration curve used for estimation of NH_3.

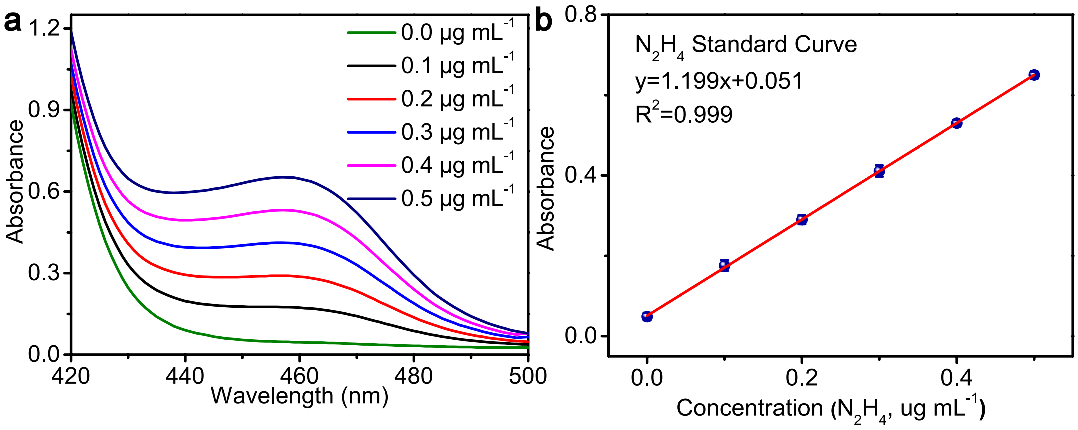

Supplementary Figure 14. (a) UV-Vis absorption spectra of various N_2H_4 concentrations after incubated for 20 min at room temperature. (b) Calibration curve used for calculation of N_2H_4 concentration.

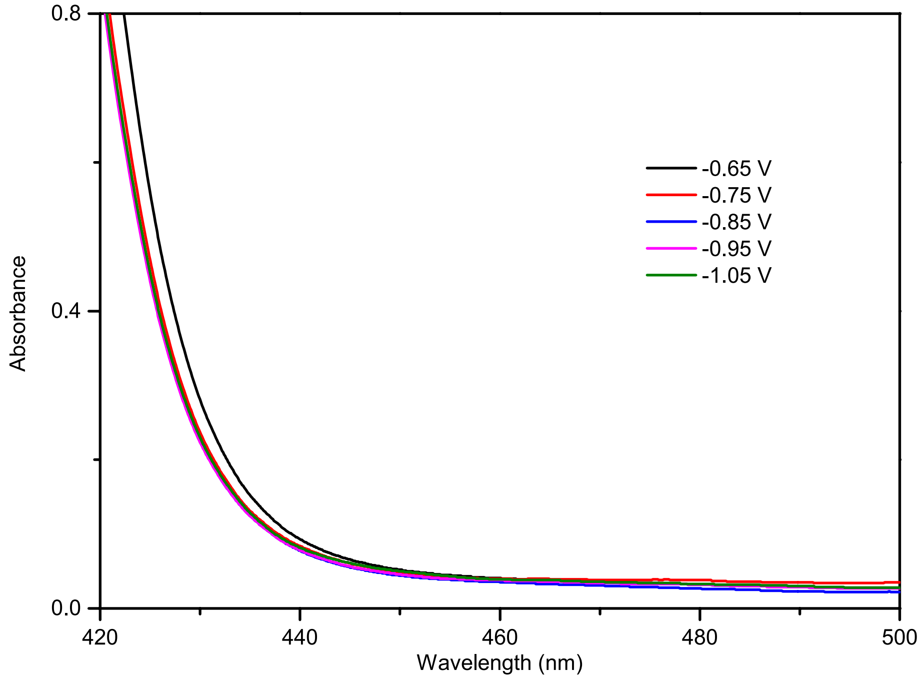

Supplementary Figure 15. UV-Vis absorption spectra of the 0.1 M Na_2SO_4 electrolytes estimated by the method of Watt and Chrisp after 2-h electrolysis in N_2 at each given potential under ambient conditions.

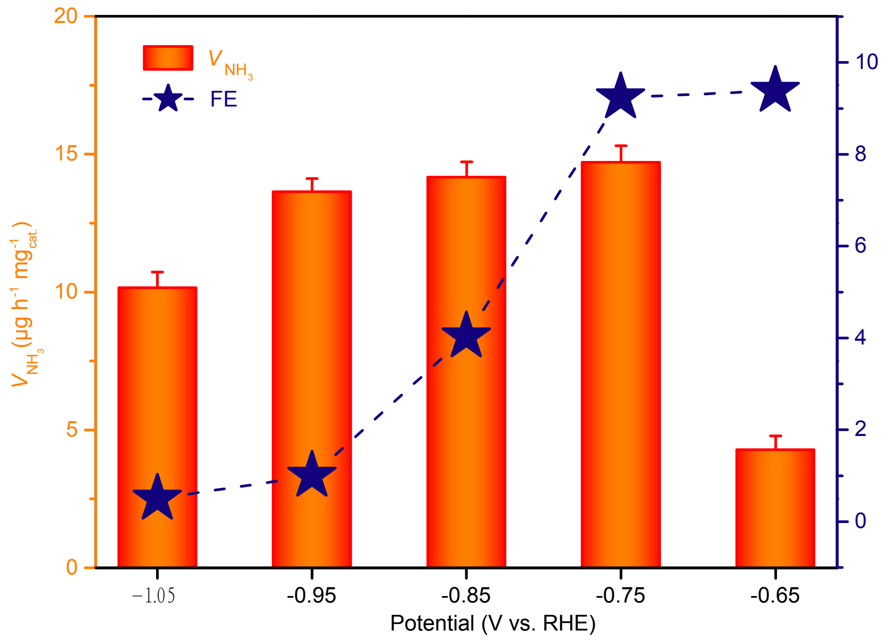

Supplementary Figure 16. NH_3 yields and FEs at each given potential in 0.1 M Na_2SO_4.

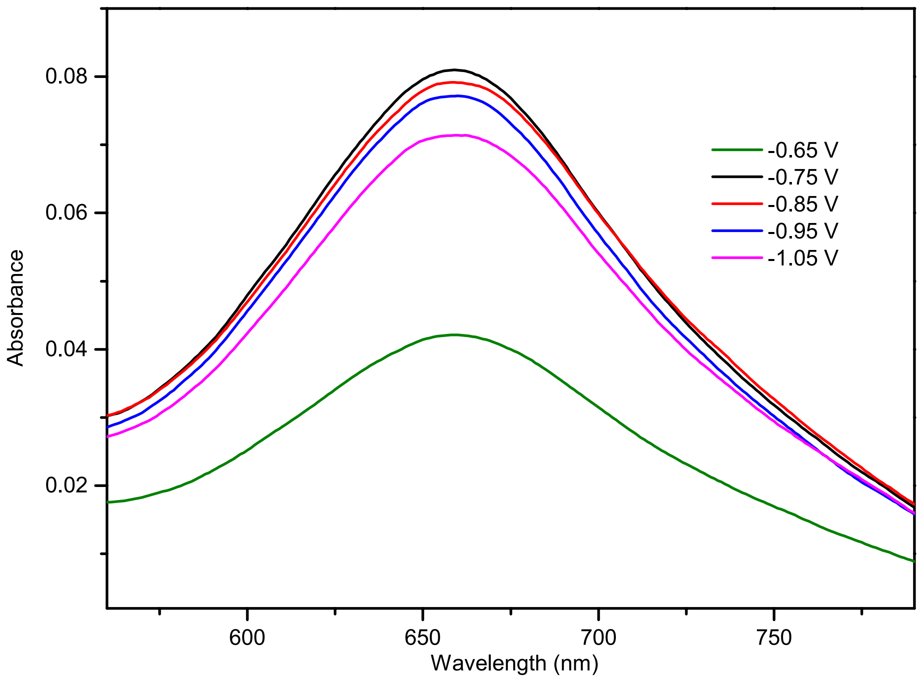

Supplementary Figure 17. UV-Vis absorption spectra of the 0.1 M Na_2SO_4 electrolytes stained with indophenol indicator after electrolysis at a series of potentials for 2 h in N_2.

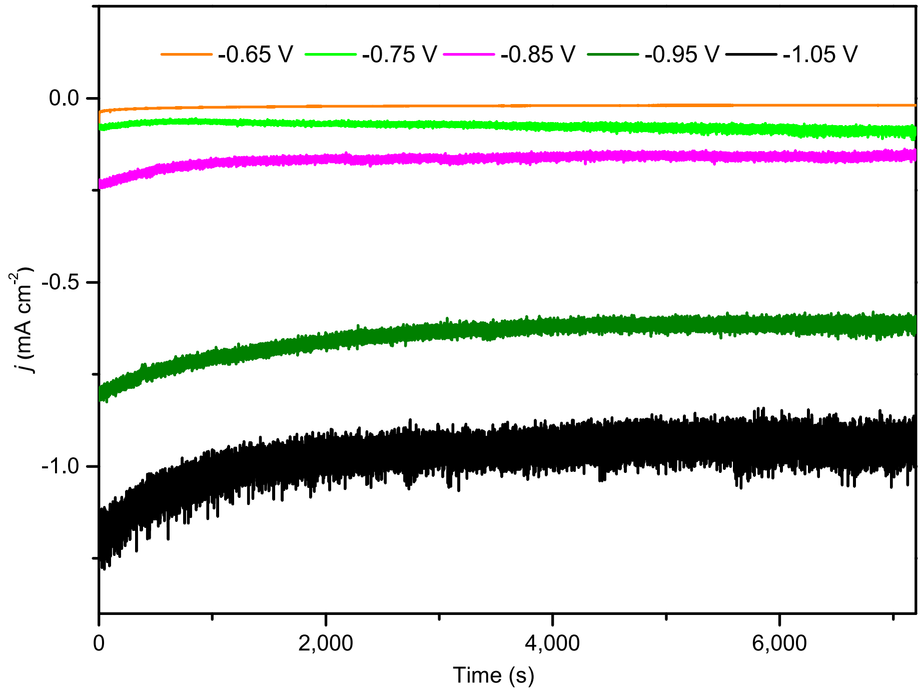

Supplementary Figure 18. Chrono-amperometry curves at a series of potentials in N_2-saturated 0.1 M Na_2SO_4.

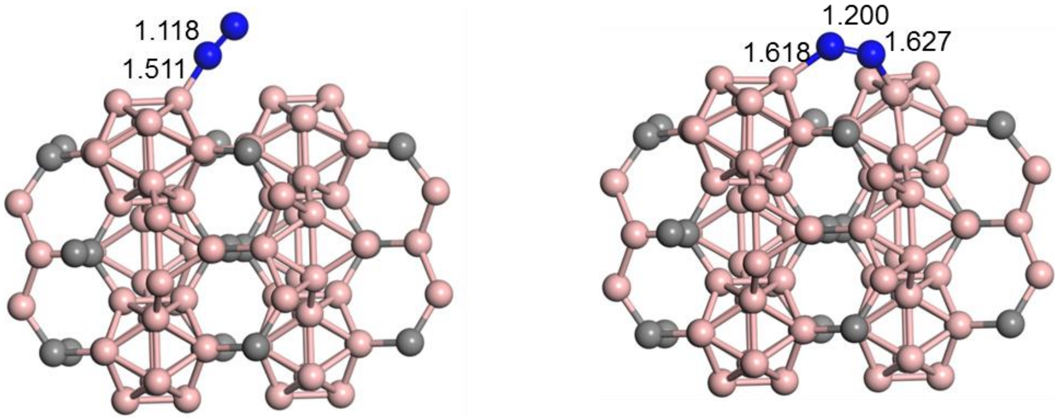

Supplementary Figure 19. Optimized structures of N_2 adsorption on the B_4C (110) surface for the end-on (left panel) and side-on (right panel) configurations. The key bond lengths (Å) are also given. Colour code: blue, N; rose, B; grey, C.

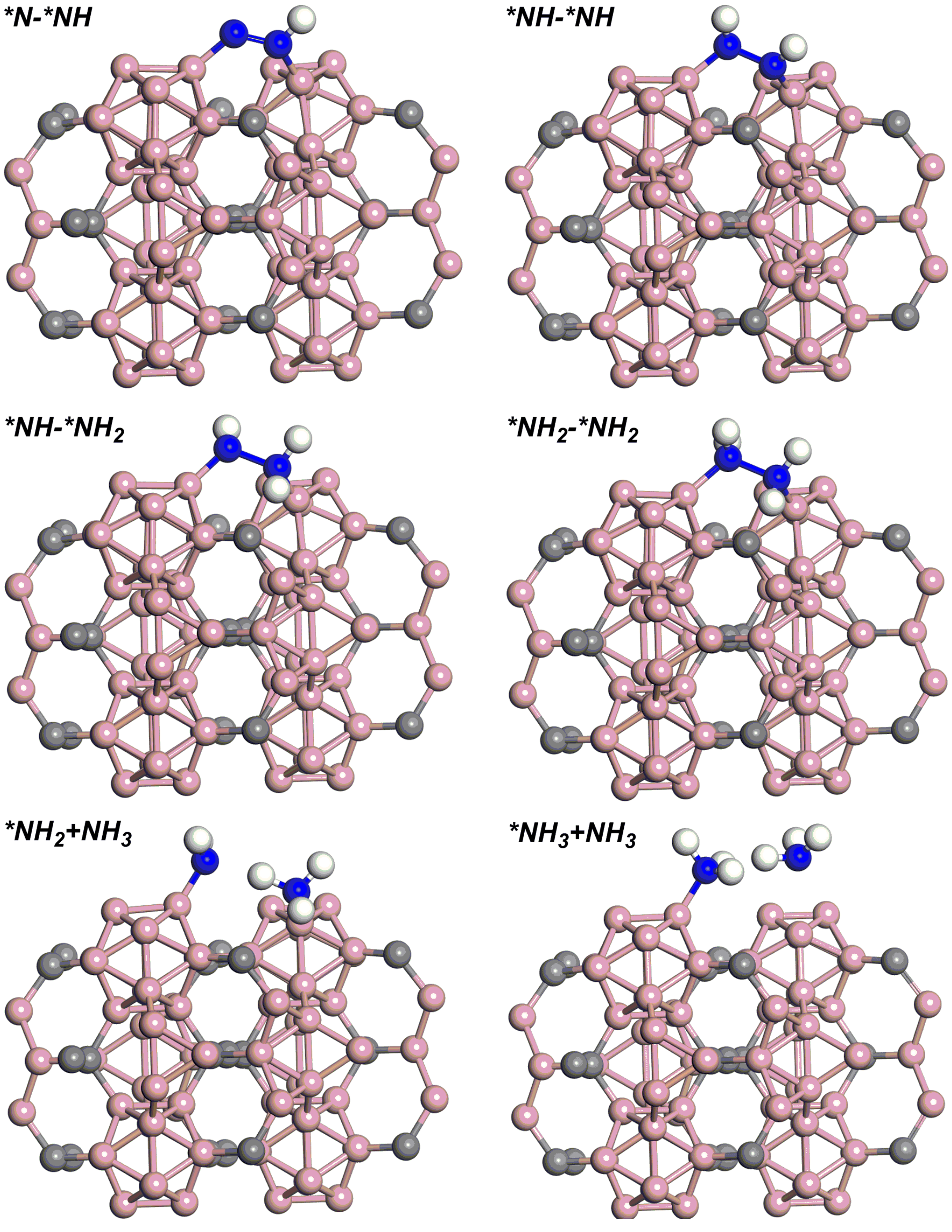

Supplementary Figure 20. Optimized geometric structures of intermediates along the reaction path proceeded on B_4C (110) surface. Colour code: blue, N; rose, B; grey, C; white, H.

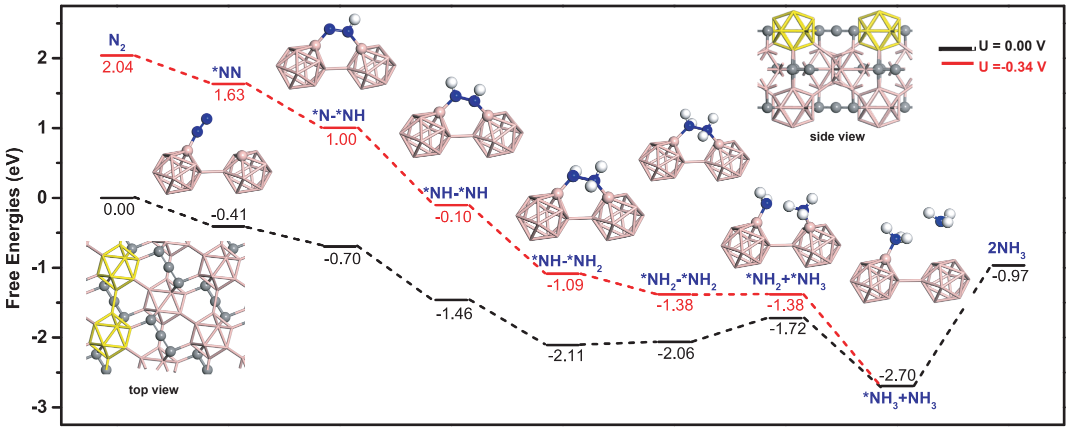

Supplementary Figure 21. Density functional theory method calculated energy profiles for the electrocatalytic N_2 fixation reaction on the B_4C (110) surface starting from the end-on adsorption structure. Colour code: blue, N; rose, B; grey, C; white, H, the asterisk * denotes an adsorption site.

Over potential and associated energy profile of the NRR: As mentioned in the main text, at potential of 0.00 V, the reaction step of $*NH_2$-$*NH_2$→$*NH_2$+$*NH_3$ is the rate-limiting step for the electrocatalytic NRR processes on the B_4C(110) surface starting from the end-on adsorption configuration. Thus, the limiting potential of –0.34 V can eliminate the free energy barrier associated with this reaction step, which is qualitatively consistent with experimental observation. Accordingly, this reaction process also becomes thermodynamically more favorable. Its free energy change varies from 0.34 eV at potential of 0.00 V to 0.00 eV at potential of –0.34 V (see black and red lines in Supplementary Fig. S21). Under this potential of –0.34 V, all electrocatalytic processes i.e. from *NN to $*NH_3$ become exothermic and thus thermodynamically favorable (see red lines). It should be noted that the free energy change of the desorption process of the NH_3 molecule is not affected because it is not an electrochemical process (still 1.73 eV at this potential of –0.34 V). However, this desorption process becomes remarkably accelerated because of much more accumulated free energy at potential of –0.34 V than at potential of 0.00 V (4.74 vs. 2.70 eV).

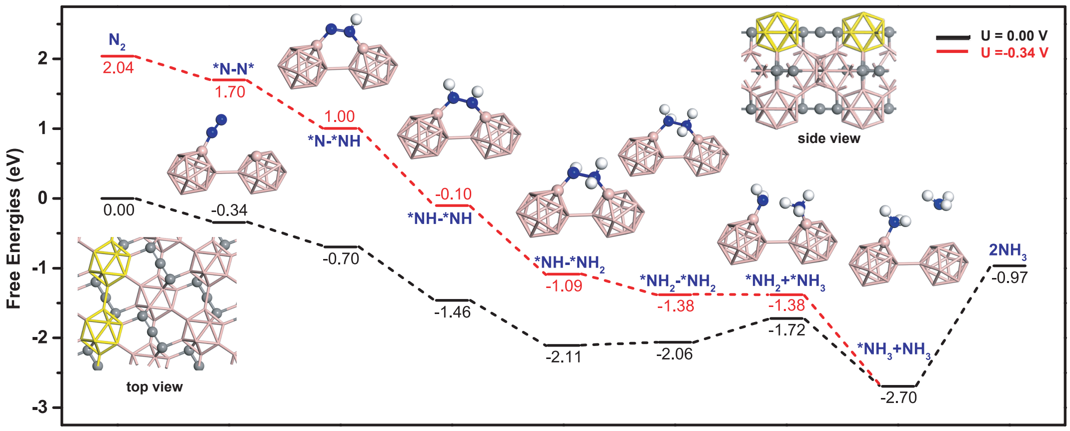

Supplementary Figure 22. Density functional theory method calculated energy profiles for the electrocatalytic NRR on the B_4C (110) surface starting from the side-on adsorption structure. Colour code: blue, N; rose, B; grey, C; white, H, the asterisk * denotes an adsorption site.

Supplementary Table 1. Comparison of electrocatalytic N_2 reduction performance for B_4C nanosheet with other electrocatalysts under ambient conditions.

Catalyst	Electrolyte	V_{NH3}	FE	Ref.
**B_4C/GCE**	**0.1 M HCl**	**26.57 μg h^{-1} mg^{-1}**	**15.95%**	**This work**
	**0.1 M Na_2SO_4**	**14.70 μg h^{-1} mg^{-1}**	**9.24%**	
N-doped porous carbon	0.05 M H_2SO_4	23.80 μg h^{-1} mg^{-1}	1.42%	1
ZIF-derived carbon	0.1 M KOH	57.8 μg h^{-1} cm^{-2}	10.20%	2
N-doped carbon nanospikes	0.25 M $LiClO_4$	97.18 μg h^{-1} cm^{-2}	11.56%	3
TA-reduced Au/TiO_2	0.1 M HCl	21.4 μg h^{-1} mg^{-1}	8.11%	4
α-Au/CeO_x-RGO	0.1 M HCl	8.31 μg h^{-1} mg^{-1}	10.1%	5
Au nanorods	0.1 M KOH	1.65 μg h^{-1} cm^{-2}	3.88%	6
AuHNCs	0.5 M $LiClO_4$	3.90 μg h^{-1} cm^{-2}	30.2%	7
Ag-Au@ZIF	THF-based electrolyte	0.61 μg h^{-1} cm^{-2}	18%	8
Ru/Ti	0.5 M H_2SO_4	7.34 μg h^{-1} cm^{-2}	-	9
Ru/C	2 M KOH	0.21 μg h^{-1} cm^{-2}	0.28%	10
Pd/C	0.1 M PBS	4.5 μg h^{-1} mg^{-1}	8.2%	11
$Bi_4V_2O_{11}$/CeO_2	0.1 M HCl	23.21 μg h^{-1} mg^{-1}	10.16%	12
MoS_2/CC	0.1 M Na_2SO_4	4.94 μg h^{-1} cm^{-2}	1.17%	13
Mo nanofilm	0.01 M H_2SO_4	1.89 μg h^{-1} cm^{-2}	0.72%	14
PEBCD/C	0.5 M Li_2SO_4	1.58 μg h^{-1} cm^{-2}	2.85%	15
Fe_2O_3-CNT	$KHCO_3$	0.22 μg h^{-1} cm^{-2}	0.15%	16
Fe-SS	Ionic liquids	1.4 μg h^{-1} cm^{-2}	60%	17

Supplementary Table 2. PBE-D computed adsorption potential energies [free energies] of the end-on and side-on adsorption configurations (Unit: eV).

	E_{ads}
end-on	0.65 [0.41]
side-on	0.63 [0.34]

Supplementary Table 3. PBE-D computed adsorption energies of the end-on adsorption configuration in the slab model with different K-points (Unit: eV).

**K-points**	**E_{ads}**
2 x 1 x 1	0.65
4 x 2 x 1	0.65

Supplementary Table 4. PBE-D computed adsorption energies of the end-on adsorption configuration in the slab models with different atomic layers (Unit: eV).

**Layers**	**E_{ads}**
three	0.65
four	0.64

Supplementary References

1. Liu, Y. et al. Facile ammonia synthesis from electrocatalytic N_2 reduction under ambient conditions on N-doped porous carbon. *ACS Catal.* **8**, 1186–1191 (2018).
2. Mukherjee, S. et al. Metal-organic framework-derived nitrogen-doped highly disordered carbon for electrochemical ammonia synthesis using N_2 and H_2O in alkaline electrolytes. *Nano Energy* **48**, 217-226 (2018).
3. Song, Y. et al. A physical catalyst for the electrolysis of nitrogen to ammonia. *Sci. Adv.* **4**, e1700336 (2018).
4. Shi, M. et al. Au sub-nanoclusters on TiO_2 toward highly efficient and selective electrocatalyst for N_2 conversion to NH_3 at ambient conditions. *Adv. Mater.* **29**, 1606550 (2017).
5. Li, S. et al*.* Amorphizing of Au nanoparticles by CeO_x–RGO hybrid support towards highly efficient electrocatalyst for N_2 reduction under ambient conditions. *Adv. Mater.* **29**, 1700001 (2017).
6. Bao, D. et al*.* Electrochemical reduction of N_2 under ambient conditions for artificial N_2 fixation and renewable energy storage using N_2/NH_3 cycle. *Adv. Mater.* **29**, 1604799 (2017).
7. Nazemi, M., Panikkanval, S. R. & El-Sayed, M. A. Enhancing the rate of electrochemical nitrogen reduction reaction for ammonia synthesis under ambient conditions using hollow gold nanocages. *Nano Energy* **49**, 316–323 (2018).
8. Lee, H. K. et al. Favoring the unfavored: selective electrochemical nitrogen fixation using a reticular chemistry approach. *Sci. Adv.* **4**, eaar3208 (2018).
9. Kugler, K., Luhn, M., Schramm, J. A., Rahimi, K. & Wessling, M. Galvanic deposition of Rh and Ru on randomly structured Ti felts for the electrochemical NH_3 synthesis. *Phys. Chem. Chem. Phys.* **17**, 3768–3782 (2015).

10. Kordali, V., Kyriacou, G. & Lambrou, C. Electrochemical synthesis of ammonia at atmospheric pressure and low temperature in a solid polymer electrolyte cell. *Chem. Commun.* **0**, 1673–1674 (2000).

11. Wang, J. et al. Ambient ammonia synthesis via palladiumcatalyzed electrohydrogenation of dinitrogen at low overpotential. *Nat. Commun.* **9**, 1795 (2018).

12. Lv, C. et al. An amorphous noble-metal-free electrocatalyst enables N_2 fixation under ambient conditions. *Angew. Chem. Int. Ed.* **57**, 6073–6076 (2018).

13. Zhang, L. et al. Electrochemical ammonia synthesis via nitrogen reduction reaction on MoS_2 catalyst: theoretical and experimental studies. *Adv. Mater.* **30**, 1800191 (2018).

14. Yang, D., Chen, T. & Wang, Z. Electrochemical reduction of aqueous nitrogen (N_2) at a low overpotential on (110)-oriented Mo nanofilm. *J. Mater. Chem. A* **5**, 18967–18971 (2017).

15. Chen, G.-F. et al. Ammonia electrosynthesis with high selectivity under ambient conditions via a Li^+ incorporation strategy. *J. Am. Chem. Soc.* **139**, 9771–9774 (2017).

16. Chen, S. et al. Electrocatalytic synthesis of ammonia at room temperature and atmospheric pressure from water and nitrogen on a carbon-nanotube-based electrocatalyst. *Angew. Chem. Int. Ed.* **56**, 2699–2703 (2017).

17. Zhou, F. et al. Electro-synthesis of ammonia from nitrogen at ambient temperature and pressure in ionic liquids. *Energy Environ. Sci.* **10**, 2516–2520 (2017).
